# Loop diuretics and subsequent use of urinary symptom medications in older adults: evaluation of a possible prescribing cascade

**DOI:** 10.1093/gerona/glaf150

**Published:** 2025-07-16

**Authors:** Matthew E Growdon, Bocheng Jing, W James Deardorff, Earl J Morris, W John Boscardin, Leah J Blank, Tasce Bongiovanni, Kenneth S Boockvar, Michael A Steinman

**Affiliations:** Division of Geriatrics, University of California, San Francisco, San Francisco, California, United States; San Francisco Veterans Affairs Medical Center, San Francisco, California, United States; Division of Geriatrics, University of California, San Francisco, San Francisco, California, United States; San Francisco Veterans Affairs Medical Center, San Francisco, California, United States; Division of Geriatrics, University of California, San Francisco, San Francisco, California, United States; San Francisco Veterans Affairs Medical Center, San Francisco, California, United States; Department of Pharmaceutical Outcomes & Policy, University of Florida College of Pharmacy, Gainesville, Florida, United States; Center for Drug Evaluation and Safety, University of Florida, Gainesville, Florida, United States; Division of Geriatrics, University of California, San Francisco, San Francisco, California, United States; Department of Epidemiology and Biostatistics, University of California, San Francisco, San Francisco, California, United States; Department of Neurology, Icahn School of Medicine at Mount Sinai, New York, New York, United States; Department of Surgery, University of California, San Francisco, San Francisco, California, United States; Division of Gerontology, Geriatrics, and Palliative Care, University of Alabama at Birmingham, Birmingham, Alabama, United States; Geriatrics Research Education and Clinical Center, Birmingham VA, Birmingham, Alabama, United States; Division of Geriatrics, University of California, San Francisco, San Francisco, California, United States; San Francisco Veterans Affairs Medical Center, San Francisco, California, United States

**Keywords:** Prescribing cascades, Prescription sequence symmetry analysis, Polypharmacy

## Abstract

**Background:**

Loop diuretic (LD) use may lead to a prescribing cascade whereby urinary symptoms are ascribed to genitourinary syndromes and treated with urinary symptom medications (USMs). We investigated if LDs lead to increased USM use among older adults and whether this potential prescribing cascade varies across key characteristics.

**Methods:**

This was a prescription sequence symmetry analysis of Veterans Administration data, involving veterans ≥66 years who initiated treatment with LD (2010-2019). USMs were antimuscarinics, beta-3 adrenergic agonists, peripheral alpha-1 blockers, and 5-alpha reductase inhibitors. We calculated the adjusted sequence ratio (aSR), assessing the cascade signal while adjusting for secular trends, and stratified by key variables.

**Results:**

There were 17 735 veterans who initiated USM within 6 months after LD and 25 190 who initiated USM within 6 months before LD; 99% were male. Unexpectedly, the aSR was 0.74 (95% CI, 0.73-0.76), meaning patients were 26% less likely to initiate USM within 6 months after initiating LD versus 6 months before. This inverse relationship held in men (aSR, 0.74, 95% CI, 0.72-0.76) but was null in women (aSR, 1.00, 95% CI, 0.80-1.26). In men without baseline urinary symptoms, we observed the LD–USM cascade in patients with heart failure (aSR 1.52, 95% CI, 1.41-1.63) and multimorbidity (eg, Charlson fourth quartile, aSR 1.24, 95% CI, 1.10-1.39).

**Conclusions:**

We did not find evidence for an LD–USM cascade among predominantly male older adults overall. Clinicians may underprescribe USMs in patients receiving LDs, perhaps due to strong attribution of urinary symptoms to LD use.

## Introduction

Loop diuretics (LDs) are a cornerstone of pharmacotherapy for a variety of health conditions prevalent in older age, such as heart failure and hypertension in the setting of chronic kidney disease.^1^ Yet, LD use is also associated with bothersome urinary symptoms, such as urinary frequency, nocturia, urgency, and potentially urinary incontinence.^2–6^ Such urinary symptoms are particularly deleterious for older adults, who may experience reduced mood, autonomy, and dignity,^7^^,^^8^ and at times avoid taking their diuretics for these reasons.^9^^,^^10^

Loop diuretics increase urine production by the kidney, and clinicians are therefore likely aware of their potential to cause bothersome urinary symptoms. Nevertheless, LD use may still lead to a prescribing cascade among older adults, whereby drug-related urinary symptoms are in turn treated with urinary symptom medications (USMs) such as bladder antispasmodics and peripheral alpha-1 blockers.^1^^,^^11^ The LD–USM prescribing cascade may occur by several mechanisms, both unintentional and intentional.^12^ For example, treating clinicians may not be aware of medication changes that occurred in other settings in which case they may be unaware of drug-related causes for urinary symptoms, leading to an unintentional prescribing cascade. Furthermore, urinary symptoms and conditions, including overactive bladder and benign prostatic enlargement (in men), are highly prevalent among older adults.^2^^,^^7^^,^^13–15^ The prevalence of lower urinary tract symptoms has been estimated to be as high as 70% in men aged 80 years or older and 90% in those 90 years or older.^13^ Given limited time during appointments, clinicians may respond reflexively to urinary symptoms by starting USMs. Alternatively, clinicians may intentionally initiate USMs to mitigate bothersome urinary symptoms when they and/or their patients have decided that maintenance of diuretic therapy (eg, to manage heart failure) is paramount, despite potential side effects.

Inquiry into the potential LD–USM prescribing cascade is important for several reasons. First, given the high prevalence of LD and USM use, we would expect this cascade to be common. Second, in the scenario of the hypothesized LD–USM prescribing cascade, the addition of USMs may be unnecessary—raising the risk of drug-related adverse effects (eg, antimuscarinics increasing risk of falls or cognitive impairment^16–18^), providing little benefit, and failing to address the root cause of the problem. Third, study of this prescribing cascade can shed light on prescribing-related behavior when clinicians are likely aware of drug-related side effects (*vs* prescribing cascades in which side effects of the initial medication are more likely to be overlooked).

Despite clinical recognition of the diuretic–USM prescribing cascade,^1^ little prior work has rigorously quantified whether diuretics lead to a prescribing cascade involving USMs among older adults.^4^ A 2022 international Delphi process identified the diuretic–overactive bladder medication cascade as one of 9 clinically important prescribing cascades, but its inclusion resulted from expert input rather than empiric evidence from a literature review.^11^ A 2024 study involving Dutch community pharmacy data reported an association between initiation of diuretics and USMs, but this was one of dozens of cascades interrogated and not the primary focus of investigation.^19^ Building on prior literature, we hypothesized that LDs lead to increased use of USMs among older veterans and sought to determine whether the potential presence of this prescribing cascade varies across key clinical and health system characteristics.

## Methods

### Design

We used prescription sequence symmetry analysis (PSSA) to assess the potential association between LD use and USM use and to identify those groups facing the highest risk of this cascade. In PSSA, the analytic cohort includes individuals who initiated 2 drugs of interest during a given observation period—a medication suspected of causing an adverse event (ie, index drug, in this case, LD) and a medication potentially used to treat the adverse event (ie, marker drug, in this case, USM). The underlying logic of PSSA is that if no causal relationship exists between use of the index drug and subsequent prescribing of the marker drug, new users of both drugs would be equally likely to receive them in either order. By contrast, in the case of a prescribing cascade, a higher proportion of initiations of marker drug would occur after the index drug compared with before.^20^ As a self-controlled study design, PSSA controls for time-invariant characteristics (eg, some demographic factors like sex) since factors that are stable over time during an observation period cannot predict the sequencing of index and marker drugs.^20^^,^^21^

### Data source

We used US Veterans Affairs (VA) outpatient pharmacy data merged with VA and Medicare claims from 2010 to 2019. This study was approved by the institutional review boards of the San Francisco VA Health Care System and the University of California, San Francisco School of Medicine and conformed to the Strengthening the Reporting of Observational Studies in Epidemiology guideline (Supplementary eTable 1).

### Study population

The study cohort included older adults receiving care in the VA who newly started an LD during the period of January 1, 2013 to August 31, 2019, and who were aged ≥ 66 years at the time of LD initiation to allow for ≥ 1 year of Medicare eligibility. We defined new users of LD as those receiving a new LD fill without any LD fills during the preceding year. The new LD fill was considered the index date. Among new users of LD, we then restricted the cohort to those individuals who newly started USM within 6 months before or after the index date. We defined new users of USM as those receiving a new USM fill without any USM fills during the preceding year. We chose this observation window to reduce time-varying confounding factors (eg, aging and disease progression) and given that 6 months is a reasonable period in which patients may present with and be treated for drug-induced urinary symptoms. Exclusion criteria included: enrollment in Medicare Advantage from 1 year to 6 months after the index date (given utilization data may not be complete during the study period^22^) and initiation of index/marker drugs on the same date in line with prior PSSA studies.^23^

### Study drugs

The index drugs were LDs, which included furosemide, torsemide, bumetanide, and ethacrynic acid. The marker drugs were USMs from the following classes: antimuscarinics, beta-3 adrenergic agonists, peripheral alpha-1 blockers, and 5-alpha reductase inhibitors (Supplementary eTable 2). Of note, we excluded prazosin within the alpha blocker class given its frequent use for posttraumatic stress disorder among veterans. We included a wide range of USMs to account for the possibility that LDs may prompt clinicians to prescribe medications for different indications (eg, for overactive bladder *vs* benign prostatic hypertrophy). To exclude very short-term or highly intermittent use, we required fills to have a dispensed quantity of 14 or more pills.

### Key variables

We collected demographic information, including age, sex, and race/ethnicity (using categories from the Research Triangle Institute definitions found in Medicare claims^24^). We determined the burden of comorbidities using the Deyo adaptation of the Charlson comorbidity index (calculated from VA and Medicare claims during the 2 years before initiation of whichever drug [index or marker] came first), which was categorized into quartiles.^25^ We determined the presence of dementia at baseline based on a 3-year look-back period in line with prior studies.^26^^,^^27^ We defined baseline chronic medication use as fills of greater than or equal to 14 pills in the 6 months prior to the index date. Since many veterans receive care from both VA and Medicare and dual use may be associated with care fragmentation and problematic prescribing,^28^^,^^29^ we characterized the prescribing source for index and marker drugs (VA only, Medicare only, or VA/Medicare). Additional healthcare utilization variables included the presence of hospitalization in the prior year and the number of outpatient clinic visits in the prior year (in both VA/Medicare data).

### Statistical analysis

We first calculated the crude sequence ratio (cSR), the effect measure of PSSA, by dividing the number of individuals with the initial marker drug claim after the initial index drug by the number of individuals with the initial marker drug claim before the index drug claim. To assess the cSR graphically, we visually inspected histograms depicting marker drug initiation relative to index drug initiation for asymmetry. To adjust for secular trends in medication use during the course of the observation period (eg, increasing use of USMs over time), we calculated the null-effect sequence ratio.^30^ The null-effect sequence ratio is an expected sequence ratio reflecting the probability of the sequencing of initiation of marker drugs after index drugs in the absence of a causal association, as may occur with temporal changes in prescribing habits for the index and/or marker drug.^20^^,^^31^^,^^32^ Finally, we calculated an adjusted sequence ratio (aSR) with 95% confidence intervals (CIs) by dividing the cSR by the null-effect ratio.^33^

Prescribing behaviors potentially giving rise to the LD–USM cascade may depend on prescribing clinicians’ awareness of baseline urinary diagnoses; as such, we conducted a subgroup analysis after excluding patients who had baseline diagnoses in the past year of benign prostatic hypertrophy, overactive bladder, incontinence, nocturia, urinary retention, polyuria, and/or functional incontinence (Supplementary eTable 3). Similarly, behaviors may depend on differences in the etiology and management of urinary symptoms between men and women. As such, we conducted sex-stratified PSSA. Subsequently, we conducted stratified analyses by comparing the aSR across key clinical and health system characteristics, selected based on prior literature.^20^^,^^21^^,^^34–36^ For stratified analyses, we calculated Cochran Q statistics to test for heterogeneity of the aSR between different levels of each variable (ie, effect measure modification). Given the simultaneous testing of approximately 20 variables, we applied the Bonferroni correction and set the significance threshold as 0.0025 (0.05/20). In the Results section, we report those strata in which we observed qualitative effect modification, meaning that we observed aSR estimates on opposite sides of 1.^37^

We used SAS, version 9.4 (SAS Institute, Inc., Cary, NC) and R version 3.6.1 (R Foundation for Statistical Computing, Vienna, Austria).

## Results

### Cohort description

After applying exclusion criteria, the overall cohort included 17 735 veterans who initiated a USM within 6 months after initiating an LD and 25 190 veterans who initiated a USM within 6 months before initiating a LD (Supplementary eFigure 1). Patients had a mean age of 79.2 years, 99% were male, 8% were non-Hispanic Black, 3% were Hispanic, and 84% were Non-Hispanic White (Table 1). Peripheral alpha-1 blockers were the most commonly initiated USM within 6 months of LD (79%), followed by 5-alpha reductase inhibitors (12%), antimuscarinics (9%), and beta-3 adrenergic agonists (1%).

**Table 1.** Analytic cohort characteristics

	**LD → USM** *N* ** = 17 735**	**USM → LD** *N* ** = 25 190**	**Total** *N* ** = 42 925**
**Age [Mean (*SD*)], years**	79.1 (8.4)	79.2 (8.4)	79.2 (8.4)
**66-74**	6352 (35.8%)	8852 (35.1%)	15 204 (35.4%)
**75-84**	5895 (33.2%)	8511 (33.8%)	14 406 (33.6%)
**85+**	5488 (30.9%)	7827 (31.1%)	13 315 (31%)
**Sex: Female**	255 (1.4%)	267 (1.1%)	522 (1.2%)
**Race/**e**thnicity^a^**			
**AAPI, American Indian/Alaska Native, unknown, other**	957 (5.4%)	1316 (5.2%)	2273 (5.3%)
**Hispanic**	440 (2.5%)	658 (2.6%)	1098 (2.6%)
**Non-Hispanic Black**	1433 (8.1%)	2029 (8.1%)	3462 (8.1%)
**Non-Hispanic White**	14 905 (84%)	21 187 (84.1%)	36 092 (84.1%)
**Charlson Comorbidity Index (quartiles), no.**			
**0-2**	4654 (26.2%)	7442 (29.5%)	12 096 (28.2%)
**3-4**	4006 (22.6%)	5836 (23.2%)	9842 (22.9%)
**5-7**	5208 (29.4%)	7082 (28.1%)	12 290 (28.6%)
**>8**	3867 (21.8%)	4830 (19.2%)	8697 (20.3%)
**Congestive heart failure**	8778 (49.5%)	9348 (37.1%)	18 126 (42.2%)
**Hypertension**	13 619 (76.8%)	19 057 (75.7%)	32 676 (76.1%)
**Renal failure**	3105 (17.5%)	3994 (15.9%)	7099 (16.5%)
**Liver disease**	1015 (5.7%)	1349 (5.4%)	2364 (5.5%)
**Venous stasis**	947 (5.3%)	1259 (5%)	2206 (5.1%)
**Dementia**	2110 (11.9%)	3110 (12.3%)	5220 (12.2%)
**Baseline medication count, no.**			
**0-4**	9412 (53.1%)	13 702 (54.4%)	23 114 (53.8%)
**5-9**	3325 (18.7%)	4678 (18.6%)	8003 (18.6%)
**10+**	4998 (28.2%)	6810 (27%)	11 808 (27.5%)
**Prescribing data source**			
**VA only**	6795 (38.3%)	9929 (39.4%)	16 724 (39%)
**Medicare only**	10 376 (58.5%)	14 326 (56.9%)	24 702 (57.5%)
**VA and Medicare**	564 (3.2%)	935 (3.7%)	1499 (3.5%)
**Hospitalization in the past year**	8327 (47%)	11 357 (45.1%)	19 684 (45.9%)
**Clinic visits in the past year ≥ median (28 visits)**	8751 (49.3%)	11 784 (46.8%)	20 535 (47.8%)
**Index year**			
**2013-2015**	9460 (53.3%)	13 439 (53.4%)	22 899 (53.3%)
**2016-2019**	8275 (46.7%)	11 751 (46.6%)	20 026 (46.7%)
**Incident USM**			
**Antimuscarinics**	1629 (9.2%)	2153 (8.5%)	3782 (8.8%)
**Beta-3 adrenergic agonists**	181 (1.0%)	216 (0.9%)	397 (0.9%)
**Peripheral alpha-1 blockers**	13 925 (78.5%)	19 808 (78.6%)	33 733 (78.6%)
**5-alpha reductase inhibitors**	2000 (11.3%)	3013 (12%)	5013 (11.7%)

Abbreviations: AAPI, Asian/Pacific Islander; LD, loop diuretic; USM, urinary symptom medication; VA, Veterans Affairs.

a Race/Ethnicity categories were assigned based on the Research Triangle Institute definitions found in Medicare claims.

### Prescription sequence symmetry analyses

**Figure 1.**
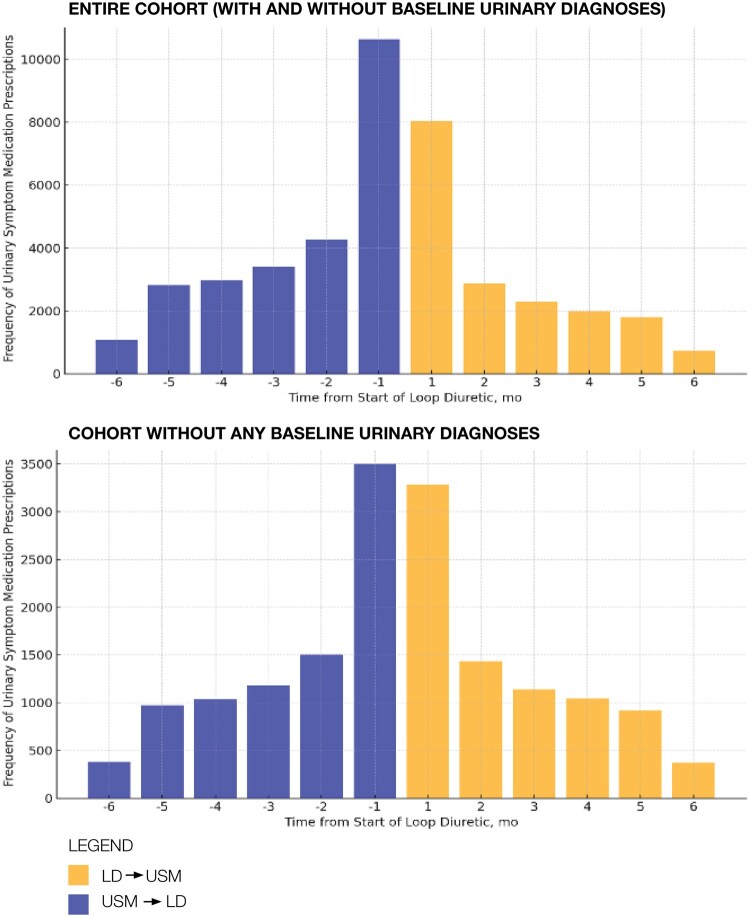
Prescription sequence symmetry of initial urinary symptom medication prescription within 6 months of initial loop diuretic prescription among older veterans, in the overall cohort (top panel) and the subgroup analysis excluding patients with baseline urinary diagnoses (bottom panel). In the absence of an association between loop diuretic and urinary symptom medication use, we would expect the pattern to be symmetrical around time 0. In the overall cohort (top panel), the relative excess volume of patients on the left-hand side of the figure (loop diuretic following urinary symptom medication) compared to the right-hand side (loop diuretic preceding urinary symptom medication) indicates an inverse prescribing order (USM–LD). In the cohort excluding baseline urinary diagnoses (bottom panel), the plot is relatively symmetric, indicating a null finding. LD, loop diuretic; USM, urinary symptom medication.

Among older veterans prescribed both an LD and USM within a 6-month period, the aSR was 0.74 (95% CI, 0.73-0.76), meaning patients were 26% less likely to receive a USM after initiating LD compared to before. Among patients with baseline urinary diagnoses, the aSR was 0.61 (95% CI, 0.59-0.63). Among patients without baseline urinary diagnoses, the aSR was attenuated to the null: 1.01 (95% CI, 0.97-1.05), meaning there was no difference in the sequencing of LD and USM. Figure 1 depicts the PSSA histograms before and after exclusion of patients with baseline urinary diagnoses.

In sex-stratified analyses, we observed a similar inverse relationship among men (aSR, 0.74, 95% CI, 0.72-0.76), whereas among women, there was a null relationship (aSR, 1.00, 95% CI, 0.80-1.26; *P* value for heterogeneity of aSRs = 0.009). Given the small number of women (*N* = 522), we limited subsequent analyses to men only.

**Figure 2.**
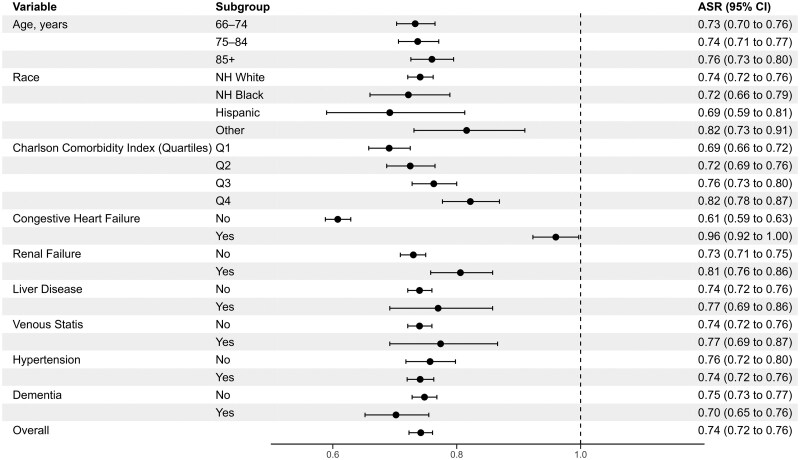
Stratified prescription sequence symmetry analysis by sociodemographic and patient factors among older male veterans. Stratified analyses should be interpreted judiciously given the risk of false positive findings with multiple hypothesis testing. aSR, adjusted sequence ratio; NH, non-Hispanic. “Other” race refers to Medicare claims categorizations of Asian/Pacific Islander, American Indian/Alaska Native, unknown, and other.

**Figure 3.**
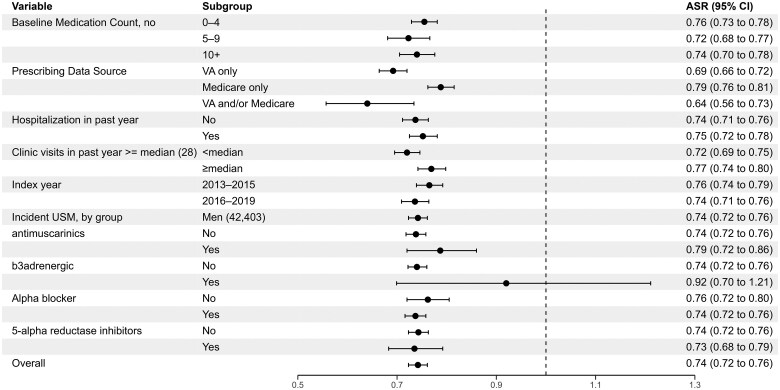
Stratified prescription sequence symmetry analysis by medication and health services factors among older male veterans. Stratified analyses should be interpreted judiciously given the risk of false positive findings with multiple hypothesis testing. aSR, adjusted sequence ratio; VA, Veterans Affairs.

Among older male veterans (*N* = 42 403), stratified analyses by key clinical and health system characteristics exhibited overlapping 95% CIs across stratum levels and estimates indicating an inverse association in almost all cases (Figures 2 and 3 and Supplementary eTable 4). In the subgroup of men excluding patients with baseline urinary diagnoses (*N* = 16 509), most stratified analyses similarly showed null relationships (Supplementary eFigures 2 and 3 and Supplementary eTable 5). However, there were several characteristics in which a LD–USM signal emerged in men without baseline urinary diagnoses: among those with a history of heart failure (aSR 1.52, 95% CI, 1.41-1.63; compared with those without a history of heart failure, aSR 0.83, 95% CI, 0.79-0.87, *p* < .001 for heterogeneity of aSRs), higher comorbidity burden (eg, Charlson comorbidity score, fourth quartile, aSR 1.24, 95% CI, 1.10-1.39; third quartile, aSR 1.12, 95% CI, 1.03-1.21; compared to second quartile, aSR 1.02, 95% CI, 0.93-1.11, first quartile, aSR 0.90, 95% CI, 0.84-0.96, *p* < .001 for heterogeneity of aSRs), at least one hospitalization in the prior year (aSR 1.15, 95% CI, 1.07–1.24 compared to no hospitalization in the prior year, aSR 0.95, 95% CI, 0.90-1.0, *p* < .001 for heterogeneity of aSRs), and greater number of clinic visits (≥ sample median of 28 visits, aSR 1.15, 95% CI, 1.07-1.24, compared to < sample median, aSR 0.95, 95% CI, 0.90-0.99, *p* < .001 for heterogeneity of aSRs).

## Discussion

Contrary to expectation, we did not find strong evidence of an LD–USM cascade among predominantly male older veterans. Rather, our overall PSSA revealed evidence for the inverse prescribing order (USM–LD), reflected by an aSR of 0.74 (95% CI, 0.73-0.76). When excluding patients with baseline urinary diagnoses, this relationship was attenuated, and we did not find any difference in USM prescribing before versus after LD initiation (aSR 1.01, 95% CI, 0.97-1.05). We observed an inverse relationship among men (aSR, 0.74, 95% CI, 0.72-0.76), whereas among the relatively small number of women in our cohort, there was a null relationship (aSR, 1.00, 95% CI, 0.80-1.26). In stratified analyses of older male veterans without baseline urinary diagnoses, we found modest LD–USM signals among older men with a history of heart failure and those with greater medical complexity.

Our findings represent important empirical evidence regarding the potential LD–USM cascade, considered one of 9 clinically important prescribing cascades based on an international expert review in 2022.^11^ Despite this designation, we are aware of only a handful of rigorous studies that have focused specifically on this cascade. Kalisch Ellett et al.^4^ conducted PSSA using Australian VA data, assessing initiation of oxybutynin before and after several medications associated with urinary incontinence, including diuretics. Like our overall findings, they reported a significant inverse association among older veterans, finding a greater risk of initiating LDs after oxybutynin (aSR 0.84, 95% CI, 0.79-0.90), which they attributed to potential unmeasured confounding.^4^ Mohammad et al. conducted a PSSA using Dutch community pharmacy data, in which they found a positive association (aSR 1.13, 95% CI, 1.06-1.21) among older adults between diuretics and urological medications (including medications used for erectile dysfunction, but not medications used for benign prostatic hypertrophy). At the same time, the authors did not find evidence of an association between diuretics and medications used in benign prostatic hypertrophy.^19^ By taking a deep dive into exploring this potential prescribing cascade, our study provides novel insights to help make sense of these discrepant findings. Variation of findings in these prior studies and our own underscore how the specifications of PSSA (eg, whether focusing on one specific drug or a class of drugs for index and/or marker drugs) have important implications for the interpretation of results.^23^

Although we lack an unambiguous explanation for our findings, several factors may have contributed to the results. We thus can only speculate regarding potential explanations for the observed findings, which would be best elucidated through qualitative inquiry into the decision-making underpinning prescribing cascades. On the one hand, the inverse finding could represent a situation in which USM use increases the risk of using LD, for example, through drug-related exacerbation of heart failure, leading to LD use. Prior studies have raised the possibility that some USMs, including antimuscarinics, beta-3 adrenergic agonists, and peripheral alpha-1 blockers, could contribute to adverse effects in heart failure, but the clinical relevance of these relationships is uncertain.^1^^,^^38^ On the other hand, LD use may indirectly reduce the risk (at a group level) of using USM through clinician and patient behavior. This scenario would occur if clinicians prescribing LDs and/or their patients recognize urinary symptoms as expected side effects from LD use and opt not to pursue pharmacological treatment of these symptoms with USMs. This scenario also raises the possibility that clinicians may be underprescribing USMs in patients receiving LDs (eg, through undertreatment of prostatic hypertrophy). It is possible that factors other than clinician behavior may have driven the results, but it is difficult to ascertain what these might be given the self-controlled nature of PSSA design, which minimizes the impact of other potential contributors such as access to healthcare.

Results from the subgroup of men excluding those with baseline urinary diagnoses warrant further discussion. This subgroup analysis was analogous to prior PSSA studies, which excluded individuals with baseline diagnoses that would represent an indication for prescribing of the marker drug. For example, prior studies have excluded individuals with diagnosed heart failure from PSSAs of the calcium channel blocker–LD and gabapentinoid–LD prescribing cascades, given that LD prescribing may be for heart failure-related fluid overload.^34–36^ We found that after excluding men with baseline urinary diagnoses, the overall aSR and most stratified aSRs were attenuated to the null, while several stratum-specific estimates emerged with LD–USM signals. Individuals with baseline urinary diagnoses had a strongly inverse SR; their exclusion thus shifted the aSR estimate closer to the null. It is possible that these individuals had previously been on USMs and were more likely to restart USM earlier in the observation period. We intended to exclude individuals with prior USM use, but some individuals may have had USM prescribed just before our exclusion period.

In the subgroup analysis of men excluding patients with baseline urinary diagnoses, there were several strata with modest evidence for the LD–USM prescribing cascade. We observed the strongest effect among men with a history of heart failure (aSR 1.52, 95% CI, 1.41-1.63), likely due to the centrality of diuretics in heart failure. Patients and clinicians are likely particularly driven in this setting to adhere to diuretic therapy to achieve symptomatic relief. This may contribute to a greater likelihood of initiating USMs to address bothersome urinary symptoms while maintaining diuretic therapy.^1^ Other important factors included greater comorbidity burden (increasing across quartiles of Charlson score), recent hospitalization, and greater number of clinic visits. A thread connecting these characteristics is the overall medical complexity of a given patient, which may lead prescribing clinicians to contribute unintentionally to a prescribing cascade. Importantly, stratified analyses are unadjusted, and therefore it is not possible to fully disentangle the interplay between factors in driving results. Although stratified analyses elucidate subgroups in whom clinicians are more or less likely to prescribe USM after LD, they preclude multivariable analyses to delineate interactions between various factors driving the potential cascade (eg, age and history of congestive heart failure). Additionally, it is important to interpret stratified analyses judiciously given the risk of false positive findings with multiple hypothesis testing.

An important limitation of our study is that findings may not be generalizable to older female patients given that VA patients are predominantly male (representing 99% of the analytic cohort), and we did not conduct further subgroup analyses among women alone due to small sample size. There are important differences in the etiology and management of urinary symptoms between men and women, which could affect the presence of the LD–USM prescribing cascade.^39^^,^^40^ Notably, we observed a null relationship among women compared to an inverse relationship (USM–LD) among men, lending credence to the notion that prescribing behaviors (including the propensity to contribute to the LD–USM prescribing cascade) may vary based on patient sex. Further exploring this question in a cohort with greater female representation is an area ripe for study.

Our study has additional limitations. First, PSSA allows detection of potential prescribing cascades at a population level by exploiting the sequencing of index and marker drugs without knowledge of the indication for prescribing or the context in which prescribing occurred.^41^ With PSSA, we are unable to determine if a prescribing cascade occurred for a given individual and, if so, whether the prescribing was potentially appropriate versus problematic for the patient or intentional versus unintentional on the part of the prescriber.^12^ Population-based studies such as PSSAs are valuable to assess overall patterns of care (ie, whether there is evidence for a cascade overall in a population) and may help direct resources and target interventions. However, such studies cannot determine the unique clinical circumstances of every individual. Importantly, other methods have been developed to determine the likelihood that an individual patient has been affected by a prescribing cascade. These include clinical process mapping and structured case review attending to the likelihood of a potential adverse drug event, its recognition by prescribers, and the health outcomes of the individual patient.^42–45^ Given the high prevalence of urinary symptoms among older men and evidence linking the use of LDs to more severe urinary symptoms, it would be sensible to consider clinical process measures capturing whether urinary symptoms worsen with LDs within individual patients to better detect potential prescribing cascades.^2^^,^^13^^,^^14^ Unfortunately, these methods cannot be operationalized in healthcare administrative data. Second, given our PSSA focused on new users of both LD and USM, we were unable to test the hypothesis that clinicians may reduce or cease LDs rather than initiating USMs. Finally, we studied a broad group of USMs targeting diverse pathophysiologies ranging from detrusor overactivity to benign prostatic hypertrophy. Although we did so given LDs may prompt clinicians to prescribe medications for different indications, this adds uncertainty to the interpretation of the study findings. Of note, stratified analyses by specific USM medication classes revealed similar findings (generally inverse findings in the overall cohort and null relationships in the subgroup of men excluding patients with baseline urinary diagnoses).

In summary, contrary to our expectation, we did not find evidence for an LD–USM cascade among a group of predominantly male older veterans; rather, older veterans were 26% less likely to receive a USM after initiating LD compared to before. Stratified analyses showed an inverse USM–LD signal across a wide range of clinical and health system variables, which was attenuated in analyses among men excluding baseline urinary diagnoses. Among older men, clinician knowledge of baseline urinary diagnoses and medical complexity is important in determining the direction and strength of this potential prescribing cascade as detected by PSSA.

## Supplementary Material

glaf150_Supplementary_Data

## Data Availability

No additional data are available for sharing owing to a data use agreement with the US Department of Veterans Affairs. The statistical code used in programming and/or analysis can be made freely available to others.
