# Supplementary material for: Loop diuretics and subsequent use of urinary symptom medications in older adults: evaluation of a possible prescribing cascade
Source: J Gerontol A Biol Sci Med Sci. 2025 Jul 16;80(8):glaf150. doi: 10.1093/gerona/glaf150 (PMC12343084; doi:10.1093/gerona/glaf150)
Supplement: glaf150_Supplementary_Data [file glaf150_supplementary_data.docx]

**Supplementary Materials**

Table of Contents

1. eTable 1: STROBE Checklist
2. eTable 2: Urinary Symptom Medications
3. eTable 3: Diagnostic Codes for Baseline Urinary Diagnoses
4. eTable 4: Stratified Prescription Sequence Symmetry Analysis by Key Clinical and Health System Characteristics (Men, With and Without Baseline Urinary Diagnoses)
5. eTable 5: Stratified Prescription Sequence Symmetry Analysis by Key Clinical and Health System (Men, Post Exclusion of Baseline Urinary Diagnoses)
6. eFigure 1: Cohort Flow Diagram
7. eFigure 2: Stratified Prescription Sequence Symmetry Analysis by Sociodemographic and Patient Factors (Subgroup of Men Excluding Patients with Baseline Urinary Diagnoses)
8. eFigure 3: Stratified Prescription Sequence Symmetry Analysis by Medication and Health Services Factors (Subgroup of Men Excluding Patients with Baseline Urinary Diagnoses)

**eTable 1: STROBE Checklist**

|  | Item No | Recommendation | Page No |
| --- | --- | --- | --- |
| **Title and abstract** | 1 | (*a*) Indicate the study’s design with a commonly used term in the title or the abstract |  |
|  |  | (*b*) Provide in the abstract an informative and balanced summary of what was done and what was found | 1-4 |
| Introduction | | | |
| Background/rationale | 2 | Explain the scientific background and rationale for the investigation being reported | 5 |
| Objectives | 3 | State specific objectives, including any prespecified hypotheses | 5 |
| Methods | | | |
| Study design | 4 | Present key elements of study design early in the paper | 6-7 |
| Setting | 5 | Describe the setting, locations, and relevant dates, including periods of recruitment, exposure, follow-up, and data collection | 6 |
| Participants | 6 | (*a*) Give the eligibility criteria, and the sources and methods of selection of participants. Describe methods of follow-up | 6 |
|  |  | (*b*) For matched studies, give matching criteria and number of exposed and unexposed |  |
| Variables | 7 | Clearly define all outcomes, exposures, predictors, potential confounders, and effect modifiers. Give diagnostic criteria, if applicable | 7-9 |
| Data sources/ measurement | 8* | For each variable of interest, give sources of data and details of methods of assessment (measurement). Describe comparability of assessment methods if there is more than one group | 7-9 |
| Bias | 9 | Describe any efforts to address potential sources of bias | 7 |
| Study size | 10 | Explain how the study size was arrived at | eFigure 1 |
| Quantitative variables | 11 | Explain how quantitative variables were handled in the analyses. If applicable, describe which groupings were chosen and why | 9-10, Table1 |
| Statistical methods | 12 | (*a*) Describe all statistical methods, including those used to control for confounding | 9-10 |
|  |  | (*b*) Describe any methods used to examine subgroups and interactions |  |
|  |  | (*c*) Explain how missing data were addressed |  |
|  |  | (*d*) If applicable, explain how loss to follow-up was addressed |  |
|  |  | (*e*) Describe any sensitivity analyses |  |
| Results | | |  |
| Participants | 13* | (a) Report numbers of individuals at each stage of study—eg numbers potentially eligible, examined for eligibility, confirmed eligible, included in the study, completing follow-up, and analysed | eFigure 1 (flow diagram) |
|  |  | (b) Give reasons for non-participation at each stage |  |
|  |  | (c) Consider use of a flow diagram |  |
| Descriptive data | 14* | (a) Give characteristics of study participants (eg demographic, clinical, social) and information on exposures and potential confounders | 11-12 |
|  |  | (b) Indicate number of participants with missing data for each variable of interest |  |
|  |  | (c) Summarise follow-up time (eg, average and total amount) |  |
| Outcome data | 15* | Report numbers of outcome events or summary measures over time | Figure 1 |

| Main results | 16 | (*a*) Give unadjusted estimates and, if applicable, confounder-adjusted estimates and their precision (eg, 95% confidence interval). Make clear which confounders were adjusted for and why they were included |  |
| --- | --- | --- | --- |
|  |  | (*b*) Report category boundaries when continuous variables were categorized |  |
|  |  | (*c*) If relevant, consider translating estimates of relative risk into absolute risk for a meaningful time period | 11-12 |
| Other analyses | 17 | Report other analyses done—eg analyses of subgroups and interactions, and sensitivity analyses | 11-12 |
| Discussion | | | |
| Key results | 18 | Summarise key results with reference to study objectives | 12-13 |
| Limitations | 19 | Discuss limitations of the study, taking into account sources of potential bias or imprecision. Discuss both direction and magnitude of any potential bias | 16-17 |
| Interpretation | 20 | Give a cautious overall interpretation of results considering objectives, limitations, multiplicity of analyses, results from similar studies, and other relevant evidence | 14-15 |
| Generalisability | 21 | Discuss the generalisability (external validity) of the study results | 16 |
| Other information | | | |
| Funding | 22 | Give the source of funding and the role of the funders for the present study and, if applicable, for the original study on which the present article is based | 18 |

*Give information separately for exposed and unexposed groups.

**Note:** An Explanation and Elaboration article discusses each checklist item and gives methodological background and published examples of transparent reporting. The STROBE checklist is best used in conjunction with this article (freely available on the Web sites of PLoS Medicine at http://www.plosmedicine.org/, Annals of Internal Medicine at http://www.annals.org/, and Epidemiology at http://www.epidem.com/). Information on the STROBE Initiative is available at http://www.strobe-statement.org.

**eTable 2: Urinary Symptom Medications**

| **Class** | **Generic Medications** |
| --- | --- |
| Antimuscarinics | solifenacin, oxybutynin, fesoterodine, darifenacin, trospium, tolterodine, flavoxate |
| Beta-3 adrenergic agonists | mirabegron, vibegron |
| Peripheral alpha-1 blockers | alfuzosin, doxazosin, terazosin, tamsulosin, silodosin |
| 5-alpha reductase inhibitors | finasteride, dutasteride |

**eTable 3: Diagnostic Codes for Baseline Urinary Diagnoses**

| **Description** | **ICD-9** | **ICD-10** |
| --- | --- | --- |
| Benign prostatic hypertrophy (BPH) | 600.XX | N40.X |
| Hypertonicity of bladder or overactive bladder | 596.51 | N32.81 |
| Urinary incontinence, unspecified | 788.30 | R32 |
| Urge incontinence | 788.31 | N39.41 |
| Mixed incontinence (male, female) | 788.33 | N39.46 |
| Stress incontinence, male | 788.32 | N39.3 |
| Incontinence without sensory awareness | 788.34 | N39.42 |
| Post void dribbling | 788.35 | N39.43 |
| Nocturnal enuresis | 788.36 | N39.44 |
| Continuous leakage | 788.37 | N39.45 |
| Overflow incontinence | 788.38 | N39.490 |
| Other urinary incontinence | 788.39 | N39.498 |
| Urinary frequency | 788.41 | R35.0 |
| Nocturia | 788.43 | R35.1 |
| Urgency of urination | 788.63 | R39.15 |
| Retention of urine, unspecified | 788.20 | R33.9 |
| Incomplete bladder emptying | 788.21 | R39.14 |
| Other specified retention of urine | 788.29 | R33.8 |
| Polyuria | 788.42 | R35.8 |
| Functional urinary incontinence | 788.91 | R39.81 |

**eTable 4:** **Stratified Prescription Sequence Symmetry Analysis by Key Clinical and Health System Characteristics (Men, With and Without Baseline Urinary Diagnoses)**

|  |  | **Entire Cohort of Men (With and Without Baseline Urinary Diagnoses) (N=42,403)** | | | | | | | |  |
| --- | --- | --- | --- | --- | --- | --- | --- | --- | --- | --- |
|  |  | **LD🡪USM** | **USM🡪LD** | **N** | **CSR** | **NSR** | **ASR** | **ASR Lower**  **95% CI** | **ASR Upper**  **95% CI** | **P-value for testing**  **heterogeneity** |
| Overall |  | 17480 | 24923 | 42403 | 0.701 | 0.946 | 0.742 | 0.723 | 0.761 | N/A |
| Data Source | VA only | 6738 | 9864 | 16602 | 0.683 | 0.988 | 0.692 | 0.664 | 0.72 | <0.001 |
|  | Medicare only | 10179 | 14132 | 24311 | 0.72 | 0.914 | 0.788 | 0.762 | 0.815 |  |
|  | VA and/or Medicare | 563 | 927 | 1490 | 0.607 | 0.949 | 0.64 | 0.557 | 0.734 |  |
| antimuscarinics | No | 16029 | 22952 | 38981 | 0.698 | 0.947 | 0.738 | 0.718 | 0.758 | 0.175 |
| antimuscarinics | Yes | 1451 | 1971 | 3422 | 0.736 | 0.935 | 0.787 | 0.72 | 0.86 |  |
| b3adrenergic | No | 17320 | 24727 | 42047 | 0.7 | 0.946 | 0.74 | 0.722 | 0.76 | 0.122 |
| b3adrenergic | Yes | 160 | 196 | 356 | 0.816 | 0.888 | 0.92 | 0.699 | 1.211 |  |
| Alpha blocker | No | 3610 | 5179 | 8789 | 0.697 | 0.915 | 0.762 | 0.72 | 0.805 | 0.297 |
| Alpha blocker | Yes | 13870 | 19744 | 33614 | 0.702 | 0.953 | 0.737 | 0.716 | 0.758 |  |
| 5-alpha reductase inhibitors | No | 15481 | 21911 | 37392 | 0.707 | 0.951 | 0.743 | 0.723 | 0.763 | 0.788 |
| 5-alpha reductase inhibitors | Yes | 1999 | 3012 | 5011 | 0.664 | 0.902 | 0.735 | 0.683 | 0.792 |  |
| Age | 66-74 | 6248 | 8758 | 15006 | 0.713 | 0.973 | 0.733 | 0.703 | 0.765 | 0.478 |
|  | 75-84 | 5805 | 8428 | 14233 | 0.689 | 0.934 | 0.737 | 0.706 | 0.771 |  |
|  | 85+ | 5427 | 7737 | 13164 | 0.701 | 0.924 | 0.76 | 0.726 | 0.795 |  |
| Race | NH White | 14691 | 20971 | 35662 | 0.701 | 0.945 | 0.741 | 0.721 | 0.762 | 0.262 |
|  | NH Black | 1406 | 2001 | 3407 | 0.703 | 0.974 | 0.722 | 0.66 | 0.789 |  |
|  | Hispanic | 433 | 649 | 1082 | 0.667 | 0.964 | 0.692 | 0.59 | 0.813 |  |
|  | Other | 950 | 1302 | 2252 | 0.73 | 0.895 | 0.816 | 0.731 | 0.91 |  |
| Charlson | Quartile 1 | 4562 | 7328 | 11890 | 0.623 | 0.901 | 0.691 | 0.658 | 0.725 | <0.001 |
|  | Quartile 2 | 3947 | 5780 | 9727 | 0.683 | 0.942 | 0.725 | 0.687 | 0.765 |  |
|  | Quartile 3 | 5145 | 7018 | 12163 | 0.733 | 0.961 | 0.763 | 0.728 | 0.8 |  |
|  | Quartile 4 | 3826 | 4797 | 8623 | 0.798 | 0.971 | 0.822 | 0.777 | 0.869 |  |
| Baseline Medication count | 0-4 | 9262 | 13539 | 22801 | 0.684 | 0.907 | 0.755 | 0.729 | 0.781 | 0.438 |
|  | 5-9 | 3286 | 4640 | 7926 | 0.708 | 0.98 | 0.723 | 0.681 | 0.766 |  |
|  | 10+ | 4932 | 6744 | 11676 | 0.731 | 0.989 | 0.74 | 0.705 | 0.776 |  |
| Hospitalization past year | No | 9262 | 13665 | 22927 | 0.678 | 0.92 | 0.737 | 0.711 | 0.763 | 0.441 |
|  | Yes | 8218 | 11258 | 19476 | 0.73 | 0.97 | 0.752 | 0.725 | 0.781 |  |
| Clinic visits in past year | <median | 8850 | 13278 | 22128 | 0.667 | 0.926 | 0.72 | 0.695 | 0.746 | 0.011 |
|  | >=median | 8630 | 11645 | 20275 | 0.741 | 0.963 | 0.769 | 0.742 | 0.798 |  |
| Index year | 2013-2015 | 9331 | 13303 | 22634 | 0.701 | 0.917 | 0.765 | 0.739 | 0.792 | 0.137 |
|  | 2016-2019 | 8149 | 11620 | 19769 | 0.701 | 0.953 | 0.736 | 0.709 | 0.764 |  |
| Dementia | No | 15405 | 21858 | 37263 | 0.705 | 0.943 | 0.748 | 0.728 | 0.768 | 0.111 |
|  | Yes | 2075 | 3065 | 5140 | 0.677 | 0.964 | 0.702 | 0.652 | 0.755 |  |
| CHF | No | 8810 | 15660 | 24470 | 0.563 | 0.925 | 0.608 | 0.588 | 0.629 | <0.001 |
|  | Yes | 8670 | 9263 | 17933 | 0.936 | 0.975 | 0.96 | 0.923 | 0.997 |  |
| Renal Failure | No | 14417 | 20956 | 35373 | 0.688 | 0.943 | 0.73 | 0.709 | 0.75 | 0.004 |
|  | Yes | 3063 | 3967 | 7030 | 0.772 | 0.958 | 0.806 | 0.758 | 0.858 |  |
| Liver Disease | No | 16473 | 23587 | 40060 | 0.698 | 0.943 | 0.74 | 0.721 | 0.76 | 0.482 |
|  | Yes | 1007 | 1336 | 2343 | 0.754 | 0.979 | 0.77 | 0.692 | 0.858 |  |
| Venous | No | 16548 | 23685 | 40233 | 0.699 | 0.944 | 0.74 | 0.721 | 0.76 | 0.445 |
|  | Yes | 932 | 1238 | 2170 | 0.753 | 0.972 | 0.774 | 0.692 | 0.866 |  |
| Hypertension | No | 4041 | 6073 | 10114 | 0.665 | 0.879 | 0.757 | 0.718 | 0.798 | 0.487 |
|  | Yes | 13439 | 18850 | 32289 | 0.713 | 0.962 | 0.741 | 0.72 | 0.763 |  |

**eTable 5:** **Stratified Prescription Sequence Symmetry Analysis by Key Clinical and Health System Characteristics (Men, Post-Exclusion of Baseline Urinary Diagnoses)**

|  |  | **Post-Exclusion of Baseline Urinary Diagnoses (N=16,509)** | | | | | | | | |  | |
| --- | --- | --- | --- | --- | --- | --- | --- | --- | --- | --- | --- | --- |
|  |  | **LD🡪USM** | **USM🡪LD** | **N** | **CSR** | **NSR** | **ASR** | **ASR Lower**  **95% CI** | **ASR Upper**  **95% CI** | **P-value for testing**  **heterogeneity** | |  |
| Overall |  | 8046 | 8463 | 16509 | 0.951 | 0.948 | 1.003 | 0.963 | 1.044 | N/A | |  |
| Data Source | VA only | 3441 | 3461 | 6902 | 0.994 | 0.998 | 0.997 | 0.937 | 1.061 | 0.927 | |  |
|  | Medicare only | 4343 | 4749 | 9092 | 0.915 | 0.903 | 1.013 | 0.96 | 1.07 |  |  |  |
|  | VA and/or Medicare | 262 | 253 | 515 | 1.036 | 1.018 | 1.017 | 0.81 | 1.277 |  |  |  |
| antimuscarinics | No | 7521 | 7911 | 15432 | 0.951 | 0.95 | 1.001 | 0.96 | 1.044 | 0.785 | |  |
| antimuscarinics | Yes | 525 | 552 | 1077 | 0.951 | 0.929 | 1.024 | 0.875 | 1.199 |  |  |  |
| b3adrenergic | No | 8002 | 8422 | 16424 | 0.95 | 0.949 | 1.002 | 0.962 | 1.043 | 0.336 | |  |
| b3adrenergic | Yes | 44 | 41 | 85 | 1.073 | 0.813 | 1.32 | 0.754 | 2.312 |  |  |  |
| Alpha blocker | No | 1313 | 1432 | 2745 | 0.917 | 0.894 | 1.026 | 0.93 | 1.132 | 0.64 | |  |
| Alpha blocker | Yes | 6733 | 7031 | 13764 | 0.958 | 0.958 | 1 | 0.957 | 1.045 |  |  |  |
| 5-alpha reductase inhibitors | No | 7302 | 7624 | 14926 | 0.958 | 0.955 | 1.003 | 0.962 | 1.046 | 0.989 | |  |
| 5-alpha reductase inhibitors | Yes | 744 | 839 | 1583 | 0.887 | 0.883 | 1.004 | 0.882 | 1.143 |  |  |  |
| Age | 66-74 | 3606 | 3780 | 7386 | 0.954 | 0.976 | 0.977 | 0.92 | 1.038 | 0.108 | |  |
|  | 75-84 | 2506 | 2739 | 5245 | 0.915 | 0.927 | 0.987 | 0.919 | 1.06 |  |  |  |
|  | 85+ | 1934 | 1944 | 3878 | 0.995 | 0.916 | 1.086 | 0.999 | 1.179 |  |  |  |
| Race | NH White | 6724 | 7023 | 13747 | 0.957 | 0.942 | 1.016 | 0.973 | 1.062 | 0.327 | |  |
|  | NH Black | 682 | 755 | 1437 | 0.903 | 0.99 | 0.912 | 0.796 | 1.045 |  |  |  |
|  | Hispanic | 223 | 255 | 478 | 0.875 | 0.993 | 0.881 | 0.695 | 1.116 |  |  |  |
|  | Other | 417 | 430 | 847 | 0.97 | 0.94 | 1.031 | 0.864 | 1.231 |  |  |  |
| Charlson | Quartile 1 | 3018 | 3805 | 6823 | 0.793 | 0.884 | 0.898 | 0.843 | 0.956 | <0.001 | |  |
|  | Quartile 2 | 1849 | 1892 | 3741 | 0.977 | 0.962 | 1.016 | 0.934 | 1.106 |  |  |  |
|  | Quartile 3 | 2026 | 1839 | 3865 | 1.102 | 0.987 | 1.116 | 1.027 | 1.213 |  |  |  |
|  | Quartile 4 | 1153 | 927 | 2080 | 1.244 | 1.006 | 1.236 | 1.103 | 1.385 |  |  |  |
| Baseline Medication count | 0-4 | 4045 | 4634 | 8679 | 0.873 | 0.895 | 0.975 | 0.922 | 1.03 | 0.183 | |  |
|  | 5-9 | 1424 | 1336 | 2760 | 1.066 | 0.994 | 1.072 | 0.972 | 1.183 |  |  |  |
|  | 10+ | 2577 | 2493 | 5070 | 1.034 | 0.999 | 1.035 | 0.962 | 1.113 |  |  |  |
| Hospitalization past year | No | 5193 | 5990 | 11183 | 0.867 | 0.914 | 0.949 | 0.904 | 0.997 | <0.001 | |  |
|  | Yes | 2853 | 2473 | 5326 | 1.154 | 1.002 | 1.151 | 1.073 | 1.236 |  |  |  |
| Clinic visits in past year | <median | 5191 | 5979 | 11170 | 0.868 | 0.917 | 0.946 | 0.901 | 0.994 | <0.001 | |  |
|  | >=median | 2855 | 2484 | 5339 | 1.149 | 0.998 | 1.151 | 1.073 | 1.236 |  |  |  |
| Index year | 2013-2015 | 4164 | 4505 | 8669 | 0.924 | 0.923 | 1.001 | 0.947 | 1.058 | 0.581 | |  |
|  | 2016-2019 | 3882 | 3958 | 7840 | 0.981 | 0.958 | 1.024 | 0.966 | 1.086 |  |  |  |
| Dementia | No | 7519 | 7972 | 15491 | 0.943 | 0.944 | 0.999 | 0.959 | 1.042 | 0.333 | |  |
|  | Yes | 527 | 491 | 1018 | 1.073 | 0.989 | 1.085 | 0.923 | 1.276 |  |  |  |
| CHF | No | 4849 | 6384 | 11233 | 0.76 | 0.915 | 0.83 | 0.79 | 0.872 | <0.001 | |  |
|  | Yes | 3197 | 2079 | 5276 | 1.538 | 1.013 | 1.518 | 1.412 | 1.633 |  |  |  |
| Renal Failure | No | 6958 | 7385 | 14343 | 0.942 | 0.943 | 0.999 | 0.957 | 1.043 | 0.592 | |  |
|  | Yes | 1088 | 1078 | 2166 | 1.009 | 0.978 | 1.032 | 0.924 | 1.153 |  |  |  |
| Liver Disease | No | 7630 | 8092 | 15722 | 0.943 | 0.944 | 0.998 | 0.958 | 1.04 | 0.207 | |  |
|  | Yes | 416 | 371 | 787 | 1.121 | 0.995 | 1.127 | 0.937 | 1.355 |  |  |  |
| Venous | No | 7715 | 8174 | 15889 | 0.944 | 0.946 | 0.998 | 0.958 | 1.039 | 0.176 | |  |
|  | Yes | 331 | 289 | 620 | 1.145 | 0.992 | 1.155 | 0.938 | 1.421 |  |  |  |
| Hypertension | No | 2534 | 3187 | 5721 | 0.795 | 0.85 | 0.936 | 0.874 | 1.002 | 0.004 | |  |
|  | Yes | 5512 | 5276 | 10788 | 1.045 | 0.987 | 1.059 | 1.008 | 1.113 |  |  |  |

**eFigure 1:** **Cohort Flow Diagram**

Exclude patients who had the same initiation dates for loop diuretic and urinary symptom medication

Overall cohort

N=42,925

Exclude patients who were on any urinary symptom medication 1 year prior to first urinary symptom medication in the +/- 180 day window

N=53,426

Exclude patients who were not on urine symptom medication 180 days prior/post LD initiation

N=181,040

Exclude patients enrolled in Medicare Advantage at any point

N=400,493

Exclude patients on loop diuretic one year prior to the index date

N=718,838

Patients age>=66 who were on loop diuretic between 2013/01/01 and 2019/08/31 from both VA and Medicare Part D data

N=1,022,304

**eFigure 2: Stratified Prescription Sequence Symmetry Analysis by Sociodemographic and Patient Factors (Subgroup of Men Excluding Patients with Baseline Urinary Diagnoses)**


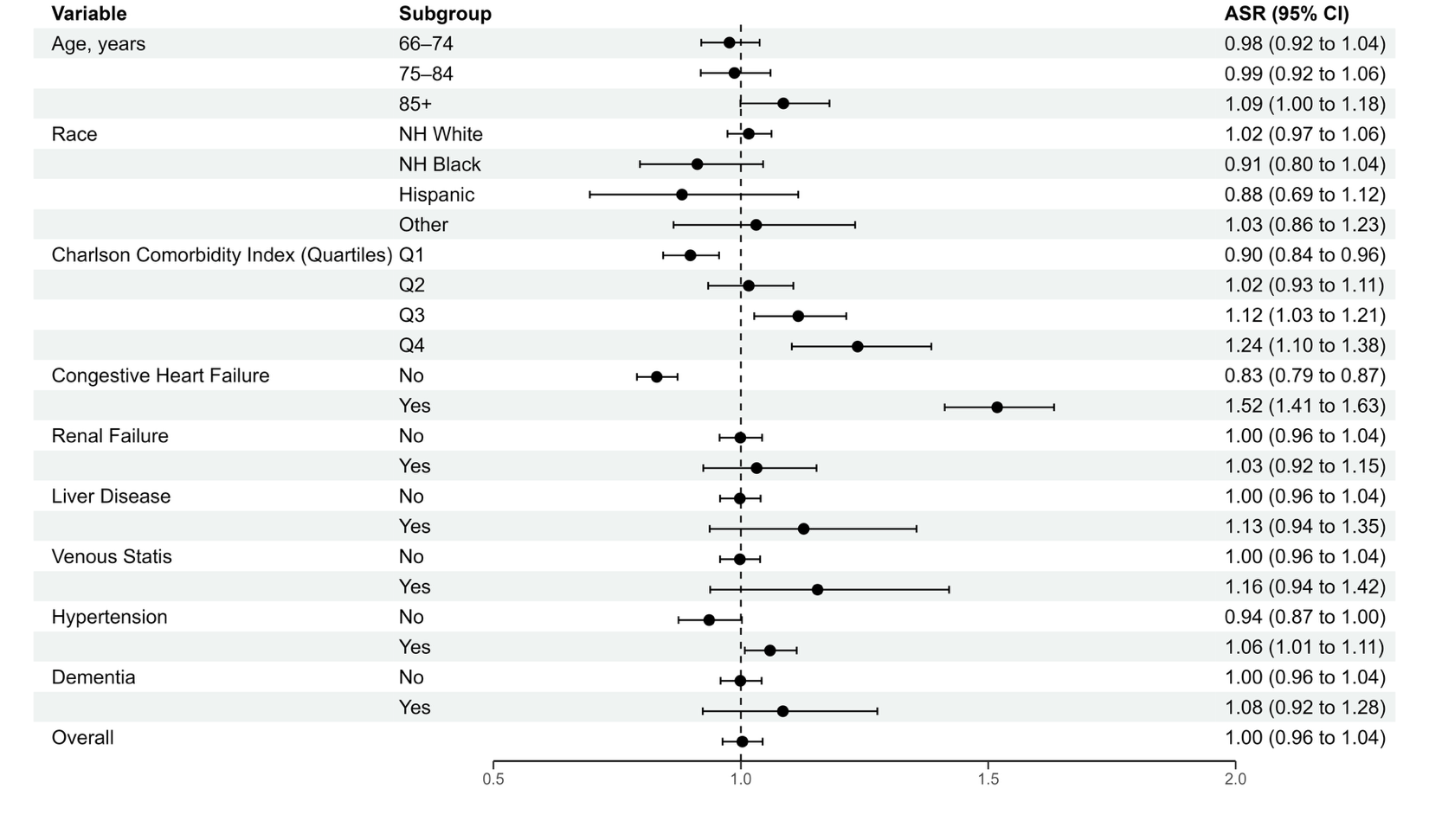


**eFigure 2 Caption**: Stratified analyses should be interpreted judiciously given the risk of false positive findings with multiple hypothesis testing. ASR = adjusted sequence ratio

**eFigure 3: Stratified Prescription Sequence Symmetry Analysis by Medication and Health Services Factors (Subgroup of Men Excluding Patients with Baseline Urinary Diagnoses)**


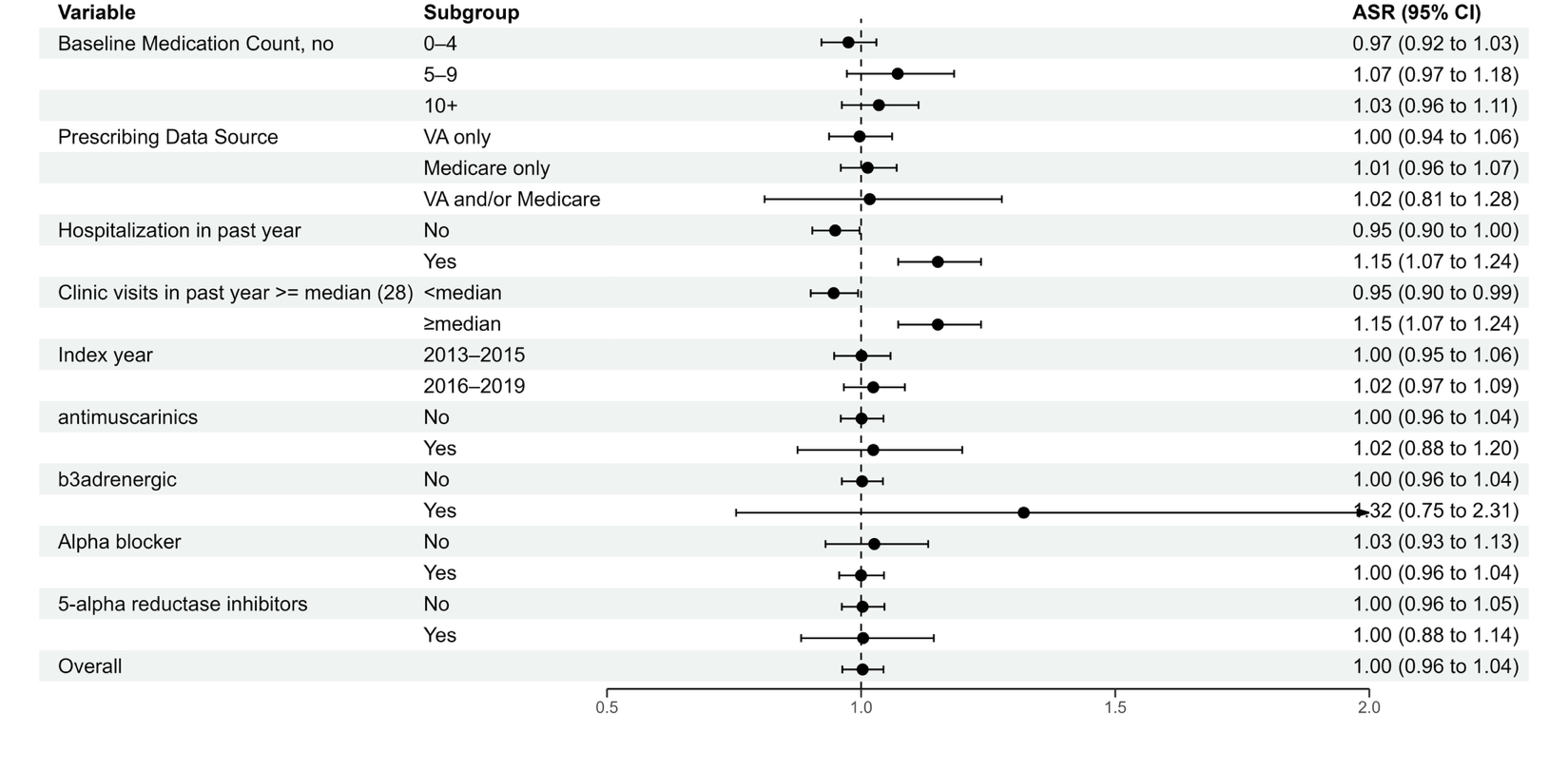


**eFigure 3 Caption**: Stratified analyses should be interpreted judiciously given the risk of false positive findings with multiple hypothesis testing. ASR = adjusted sequence ratio
